# The connection between climate change and perinatal mental health

**DOI:** 10.3389/fpsyt.2024.1515895

**Published:** 2025-01-21

**Authors:** Jennifer L. Barkin, Sanne van Rhijn, Chloe M. Johnson

**Affiliations:** ^1^ Department of Community Medicine, Mercer University School of Medicine, Macon, GA, United States; ^2^ Department of Obstetrics and Gynecology, Mercer University School of Medicine, Macon, GA, United States; ^3^ Perinatal Mental Health Service, West London National Health Service Trust, London, United Kingdom; ^4^ Department of Brain Sciences, Imperial College, London, United Kingdom

**Keywords:** climate change, perinatal mental health, extreme weather, maternal mental health, heat, Hurricane Helene

## Abstract

Climate change and extreme weather events are particularly concerning for pregnant and postpartum women and have been related to negative birth outcomes. However, the impact of climate change on perinatal mental health outcomes is not well studied. Mood and anxiety disorders are among the leading comorbidities during pregnancy and the postpartum period, and they are associated with significant familial and societal burdens. It is crucial to include environmental factors in the risk profile of perinatal mental illness to optimize prevention and early intervention strategies. In the clinical experience of one of the authors, new mothers can feel particularly concerned about their baby’s physical health when faced with extreme heat or may present as agitated due to heat-related sleep deprivation. This is in line with qualitative research showing maternal worrying about a baby’s thermal dysregulation as one of the emerging themes. With extreme weather events becoming more frequent, clinicians have a role in advocating for climate adaptation in healthcare settings. Climate inequalities need to be addressed alongside health and social inequalities.

## Introduction

Perinatal Mood and Anxiety Disorders (PMADs) are among the most common complications of pregnancy and the postpartum period but are often overlooked or untreated (1, 2). Improved prevention and detection of PMADs is crucial because untreated PMADs are associated with poorer outcomes for women and their babies, as well as considerable societal costs (3–5). Within the wide range of protective and risk factors influencing the development of PMADs, the risks posed by climate change to perinatal mental health deserve particular consideration.

## Effects of climate change on pregnancy

The most recent report by the Intergovernmental Panel on Climate Change (IPCC) indicates that the global surface temperature is likely to rise to 1.5°C above pre-industrial levels in the near term, further increasing risks to ecosystems and humans (6). With extreme heat events becoming more frequent, pregnant women and babies are considered particularly at risk of overheating. During pregnancy, a women’s capacity to lose heat is reduced because of the body shape changing and an increase in internal heat production due to fetal growth and metabolism (7). New-born babies are especially at risk because of their limited capacity for thermal regulation (7). Extreme heat exposure has consistently been associated with adverse birth outcomes such as reduced gestational age and reduced birth weight, which in turn can affect neurodevelopmental outcomes (8–10).

## Environmental injustice and maternal mental health

The effects of climate change on pregnancy outcomes are particularly evident in low- and middle-income countries. Firstly, women are more likely to be engaged in farming or other physical labor until the end of pregnancy contributing to the risk of overheating (7). Secondly, climate change related weather impacts have an immediate effect on crop production, access to clean drinking water and proper sanitation, increasing the risks of malnutrition, dehydration and infectious diseases, especially vector-borne diseases, in pregnancy (7). Indigenous and rural communities globally, relying on natural resources for their livelihood, will increasingly be impacted by climate change and face displacement. This puts communities at risk of losing cultural traditions and beliefs, negatively affecting mental health (11). An intersection of climate and social vulnerabilities is also seen in high income countries. A US government report concluded that disadvantaged groups with lower income or of minority background are more likely to live in areas with the highest projected impact of climate change driven changes in extreme temperatures (12). Pre-existing disparities in access to healthcare or climate adaptation strategies may further pronounce the relationship between climate change related weather events and birth outcomes. A review study concluded that minority and low income populations are less likely to evacuate in case of a natural disaster due to lack of transportation or finances, encountering barriers in accessing healthcare (13). Lack of financial resources may constrain a pregnant women’s access to a cooler, e.g. air-conditioned, environment.

## Extreme weather and perinatal mental health

Extreme weather events (EWEs) related to global warming, such as flash floods and heatwaves, will occur more often. Emotional distress or trauma after losses or damages due to extreme weather events will increasingly contribute to the development of PMADs (14, 15).

Literature on the relationship between rising temperatures and mental health during pregnancy and the postnatal period remains limited. One study found an increased frequency of emergency department visits among pregnant women after acute heat exposure for any psychiatric disorder, particularly suicidal thoughts (16). For PMADs, a 20% increased risk was observed after a period with several consecutive hot days, with urban populations found to be at further elevated risk (17).

To understand the relationship between extreme heat exposure and perinatal mental health in more detail, a recent study explored the views of new mothers in Australia regarding climate change or EWEs and their mental health. Emerging topics included a general sense of feeling overwhelmed by the challenges of managing extreme weather alongside motherhood, feelings of helplessness, difficulty accessing green space in an increasingly hostile climate, and worries about the implications of heat exposure on the child’s health, such as dehydration or increased body temperature (18).

While there is a growing body of evidence of climate change impacts on pregnancy and birth outcomes, the focus has been less so on mental health (7, 8). Screening for PMADs in relation to climate change does not appear to be part of regular practice yet within maternity services or programs for maternal and child health and specific interventions for climate change related PMADs are yet to be developed.

## Clinical cases

Specifically related to the clinical experience of one of the authors, parents can find it particularly challenging to settle a newborn expressing discomfort from extreme heat. This can affect a parent’s sleep and, subsequently, their ability to care for their newborn in a sensitive and emotionally attuned manner. This was the case for a woman seen for an early postnatal mental health review during a heatwave. She reported feeling exhausted because her baby had woken up every two hours the night before the review due to the heat and lack of air-conditioning. She described fleeting intrusive thoughts of smothering her baby, which evoked a strong sense of “mom guilt” and suicidal ideations. In another case, a woman was seen for a mental state review after readmission to a maternity ward in the early postnatal period where she presented with agitated behavior. The mother stated that she had become irritable with staff after her baby had been crying excessively throughout the previous nights while they had stayed in an extremely hot room. Ward staff acknowledged the role of heat in her presentation. This second case highlights how hot weather not only affects mental health but can also cause critical disruptions to vital infrastructures such as maternity hospitals. An analysis of overheating incidents in the National Health Service (NHS) in the United Kingdom, where the maximum temperature recorded on patient wards exceeded 26°C, revealed that the number of overheating incidents had doubled between 2016 and 2022 (18). In the home situation, poor families may be at particular risk of overheating due to lack of air condition or poor ventilation.

In addition to direct effect such as experiences of trauma and loss, there are indirect mental health consequences of climate change. For example, heat exposure has been associated with preterm birth or low birth weight, which in turn increases the likelihood of depression in the early postnatal period (19). Another example of the indirect impact on mental health comes from a study conducted in three South Asian countries, which found that high ambient temperature was associated with a mean increase in intimate partner violence prevalence, widely seen as a risk factor for poor perinatal mental health (20, 21). Another indirect mental health impact of global warming and related EWEs such as drought or floods is displacement or migration (20). Increasing numbers of people are migrating as a consequence of climate change (20). Refugee or migrant women are particularly vulnerable as they may already have been confronted with direct loss or trauma in their home country and then struggle with lack of access to medical care, housing stability, financial resources or social support impacting their mental health, A review found that one in three migrant women low- and middle-income countries experience depression during pregnancy or postnatally (22).

## A recent example: Hurricane Helene

Hurricane Helene, a Category 4 hurricane that devastated the south-eastern United States in late September 2024, provides a recent illustration of the impact of climate change on pregnant and postpartum women. Helene has been called the “deadliest mainland hurricane since Katrina in 2005” and caused an estimated $30.5 to $47.5 billion in damages in the United States (23, 24) Among the consequences, were weeks-long power outages, flooding, downed trees, property damage, and shortages of essential infant items such as diapers and formula. While diaper banks and breastfeeding coalitions mobilized quickly to support the shortages, there were reports from parents who were unable to feed their babies for up to 24 hours (25). The ability to provide food and safety for one’s children is a core component of maternal identity and functioning and this was compromised for many families during Hurricane Helene (26). As EWEs become more frequent and intense, families will continue to contend with distressing circumstances surrounding caregiving. A review of literature after Hurricane Katrina showed that marginalised communities reported higher and more persistent levels of psychosocial distress, depression and PTSD, exacerbated by an experience of systemic neglect or adequate response to the EWE from the authorities. Resilience was reported through mutual support and spirituality (27).

## Conclusions

Predisposing risks often disproportionately affect marginalized communities and increase pre-existing health inequalities. For example, this may relate to poorly ventilated home conditions, lack of tree canopies, and urban heat islands. Healthcare practitioners have a role in educating pregnant and lactating women of the physical and mental health risks of climate change related EWE’s and promoting a healthy lifestyle addressing environmental risks. However, more training is required to grow awareness among healthcare workers of these risks. Clinicians can also play a role in advocating for population health actions, such as promoting climate adaptation in healthcare settings or improving housing quality and better access to green spaces for cooling, which will co-benefit the emotional and physical well-being of women and birthing people during the perinatal period. Future research is needed to better understand the psychosocial and physiological mechanisms contributing to PMADs and impulsive and suicidal behaviors during extreme heat.

## Data Availability

The original contributions presented in the study are included in the article/supplementary material. Further inquiries can be directed to the corresponding author.

## References

[1] Howard LM Molyneaux E Dennis CL Rochat T Stein A Milgrom J. Non-psychotic mental disorders in the perinatal period. Lancet. (2014) 384:1775–88. doi: 10.1016/S0140-6736(14)61276-9 25455248

[2] Lee-Carbon L Nath S Trevillion K Byford S Howard LM Challacombe FL. Mental health service use among pregnant and early postpartum women. Soc Psychiatry Psychiatr Epidemiol. (2022) 57:2229–40. doi: 10.1007/s00127-022-02331-w PMC9636080 35902425

[3] Bauer A Parsonage M Knapp K Iemmi V Adelaja B Hogg S. The costs of perinatal mental health problems. London: Centre Ment Health. (2014) 192:83–90.

[4] Bar Zaken S. Folktales in Assistance of Cross-culture Therapy: Cultural mental prototype of motherhood in Russian folktales. J Poetry Therapy. (2020) 33(4):236–51.

[5] Stein A Pearson RM Goodman SH Rapa E Rahman A McCallum M. Effects of perinatal mental disorders on the fetus and child. Lancet. (2014) 384:1800–19. doi: 10.1016/S0140-6736(14)61277-0 25455250

[6] IPCC. AR6 synthesis report: Climate change 2023 (2023). Available online at: https://www.ipcc.ch/report/sixth-assessment-report-cycle/ (Accessed January 07, 2025).

[7] Rylander C Odland JØ Sandanger TM. Climate change and the potential effects on maternal and pregnancy outcomes: an assessment of the most vulnerable–the mother, fetus, and newborn child. Glob Health Action. (2013) 6:19538. doi: 10.3402/gha.v6i0.195386 23481091 PMC3595418

[8] Chersich MF Pham MD Areal A Haghighi MM Manyuchi A Swift CP. Associations between high temperatures in pregnancy and risk of preterm birth, low birth weight, and stillbirths: systematic review and meta-analysis. BMJ. (2020) 371:m3811. doi: 10.1136/bmj.m3811 33148618 PMC7610201

[9] Kidd SA Gong J Massazza A Bezgrebelna M Zhang Y Hajat S. Climate change and its implications for developing brains. J Climate Change Health. (2023) 13:100258.

[10] Veenema RJ Hoepner LA Geer LA. Climate change-related environmental exposures and perinatal and maternal health outcomes in the U.S. Int J Environ Res Public Health. (2023) 20:1662. doi: 10.3390/ijerph20031662 36767030 PMC9914610

[11] Jantarasami LC Novak R Delgado R Marino E McNeeley S. Tribes and indigenous peoples. In: impacts, risks, and adaptation in the United States: fourth national climate assessment. Washington DC, USA: National Climate Assessment (2018). p. 572–603. doi: 10.7930/NCA4.2018.CH15

[12] EPA. Climate change and social vulnerability in the United States: a focus on six impacts. Washington DC: U.S. Environmental Protection Agency (2021). Available at: www.epa.gov/cira/social-vulnerability-report. (Accessed December 06, 2025).

[13] Benevolenza MA DeRigne LA. The impact of climate change and natural disasters on vulnerable populations: a systematic review of literature. J Hum Behav Soc Environment. (2019) 2:266–81. doi: 10.1080/10911359.2018.1527739

[14] Pardon MK Dimmock J Chande R. Mental health impacts of climate change and extreme weather events on mothers. Eur J Psychotraumatol. (2024) 15:2296818. doi: 10.1080/20008066.2023.2296818 38224060 PMC10791077

[15] Rothschild J Haase E. Women's mental health and climate change Part II: Socioeconomic stresses of climate change and eco-anxiety for women and their children. Int J Gynaecol Obstet. (2023) 160:414–20. doi: 10.1002/ijgo.14514 36254375

[16] Runkle JD Sugg MM Berry A Reed C Cowan K Wertis L. Association of psychiatric emergency visits and warm ambient temperature during pregnancy: A time-stratified case-crossover study. Environ Health Perspect. (2024) 132:67001. doi: 10.1289/EHP13293 38829735 PMC11166382

[17] Radua J De Prisco M Oliva V Fico G Vieta E Fusar-Poli P. Impact of air pollution and climate change on mental health outcomes: an umbrella review of global evidence. World Psychiatry. (2024) 23:244–56. doi: 10.1002/wps.21219 PMC11083864 38727076

[18] Round our way NHS Overheating. NHS overheating in the UK at its highest rate ever. Edinburgh, UK: Round Our Way (2023).

[19] Vigod SN Villegas L Dennis CL Ross LE. Prevalence and risk factors for postpartum depression among women with preterm and low-birth-weight infants: a systematic review. BJOG. (2010) 117:540–50. doi: 10.1111/j.1471-0528.2009.02493.x 20121831

[20] Zhu Y He C Bell M. Association of ambient temperature with the prevalence of intimate partner violence among partnered women in low- and middle-income South Asian countries. JAMA Psychiatry. (2023) 80:952–61. doi: 10.1001/jamapsychiatry.2023.1958 PMC10308303 37379013

[21] Flach C Leese M Heron J Evans J Feder G Sharp D. Antenatal domestic violence, maternal mental health and subsequent child behaviour: a cohort study. BJOG. (2011) 118:1383–91. doi: 10.1111/j.1471-0528.2011.03040.x 21692968

[22] Fellmeth G Fazel M Plugge E. Migration and perinatal mental health in women from low- and middle-income countries: a systematic review and meta-analysis. BJOG. (2017) 124:742–52. doi: 10.1111/1471-0528.14184 27320110

[23] Available online at: https://abcnews.go.com/US/live-updates/hurricane-helene/?id=113931821 (Accessed January 07, 2025).

[24] Property damage from Hurricane Helene could cost owners more than $47 billion. ATL: CNN Business. (2024).

[25] Available online at: https://www.theguardian.com/us-news/2024/oct/11/hurricane-helene-formula-crisis (Accessed January 07, 2025).

[26] Barkin JL Wisner KL Bromberger JT Beach SR Terry MA Wisniewski SR. Development of the Barkin index of maternal functioning. J Women's Health. (2010) 12:2239–46. doi: 10.1089/jwh.2009.1893 PMC3003914 21054183

[27] Hamilton-Mason J Everett J J Hall C Harden S Lecloux M Mancini S. Hope floats: African American women's survival experiences after Katrina. J Hum Behav Soc Environ. (2012) 22:479–99. doi: 10.1080/10911359.2012.664982

